# Supplementary material for: An open-source framework for large-scale, flexible evaluation of biomedical text mining systems
Source: J Biomed Discov Collab. 2008 Jan 29;3:1. doi: 10.1186/1747-5333-3-1 (PMC2276192; doi:10.1186/1747-5333-3-1)
Supplement: Additional file 1 — Evaluation platform source code. The additional file contains the software evaluation framework discussed in this paper. The most current release can be downloaded from SourceForge.net [1]. See the accompanying README file for instructions on installation and use. Also included in the distribution is a collection of UIMA wrappers for some commonly used BioNLP tools and annotated corpora. [file 1747-5333-3-1-S1.zip › bionlp_uima_repository-1.0.1/docs/api/src-html/edu/uchsc/ccp/uima/ae/util/converter/AnnotatorConverter_AE.html]

```
001    package edu.uchsc.ccp.uima.ae.util.converter;
002    
003    import java.util.Arrays;
004    import java.util.HashSet;
005    import java.util.Iterator;
006    import java.util.Set;
007    
008    import org.apache.uima.analysis_engine.ResultSpecification;
009    import org.apache.uima.analysis_engine.annotator.AnnotatorConfigurationException;
010    import org.apache.uima.analysis_engine.annotator.AnnotatorContext;
011    import org.apache.uima.analysis_engine.annotator.AnnotatorContextException;
012    import org.apache.uima.analysis_engine.annotator.AnnotatorInitializationException;
013    import org.apache.uima.analysis_engine.annotator.AnnotatorProcessException;
014    import org.apache.uima.analysis_engine.annotator.JTextAnnotator_ImplBase;
015    import org.apache.uima.jcas.JCas;
016    
017    import edu.uchsc.ccp.uima.annotation.CCPAnnotator;
018    import edu.uchsc.ccp.uima.annotation.CCPTextAnnotation;
019    
020    /**
021     * Converts the annotator of an annotation based on its current annotator. This is useful if you want to combine annotations from numerous annotators
022     * to be under a single annotator.
023     * 
024     * @author williamb
025     * 
026     */
027    public class AnnotatorConverter_AE extends JTextAnnotator_ImplBase {
028    
029        public static final String PARAM_ANNOTATOR_ID = "AnnotatorID";
030    
031        public static final String PARAM_FIRST_NAME = "FirstName";
032    
033        public static final String PARAM_LAST_NAME = "LastName";
034    
035        public static final String PARAM_AFFILIATION = "Affiliation";
036    
037        public static final String PARAM_ANNOTATOR_IDS_TO_CONVERT = "AnnotatorIDsToConvert";
038        
039        private int annotatorID = -1;
040    
041        private String firstName = null;
042    
043        private String lastName = null;
044    
045        private String affiliation = null;
046    
047        private Set<Integer> annotatorIDsToConvert;
048        
049        @Override
050        public void initialize(AnnotatorContext ac) throws AnnotatorInitializationException, AnnotatorConfigurationException {
051            /* read in input parameters */
052            try {
053                annotatorID = ((Integer) ac.getConfigParameterValue(PARAM_ANNOTATOR_ID)).intValue();
054                firstName = (String) ac.getConfigParameterValue(PARAM_FIRST_NAME);
055                lastName = (String) ac.getConfigParameterValue(PARAM_LAST_NAME);
056                affiliation = (String) ac.getConfigParameterValue(PARAM_AFFILIATION);
057                annotatorIDsToConvert = new HashSet<Integer>(Arrays.asList((Integer[]) ac.getConfigParameterValue(PARAM_ANNOTATOR_IDS_TO_CONVERT)));
058                
059                if (firstName == null) {
060                    firstName = "";
061                }
062                if (lastName == null) {
063                    lastName = "";
064                }
065                if (affiliation == null) {
066                    affiliation = "";
067                }
068                    
069            } catch (AnnotatorContextException e) {
070                e.printStackTrace();
071            }
072            super.initialize(ac);
073        }
074    
075        /**
076         * cycle through all annotations and set the annotation set
077         */
078        public void process(JCas jcas, ResultSpecification arg1) throws AnnotatorProcessException {
079    
080            /* create an annotation set list */
081            CCPAnnotator ccpAnnotator = new CCPAnnotator(jcas);
082            ccpAnnotator.setAnnotatorID(annotatorID);
083            ccpAnnotator.setAffiliation(affiliation);
084            ccpAnnotator.setFirstName(firstName);
085            ccpAnnotator.setLastName(lastName);
086    
087            Iterator annotIter = jcas.getJFSIndexRepository().getAnnotationIndex(CCPTextAnnotation.type).iterator();
088    
089            while (annotIter.hasNext()) {
090                Object possibleAnnot = annotIter.next();
091                if (possibleAnnot instanceof CCPTextAnnotation) {
092                    CCPTextAnnotation ccpTA = (CCPTextAnnotation) possibleAnnot;
093                    int currentAnnotatorID = ccpTA.getAnnotator().getAnnotatorID();
094                    if (annotatorIDsToConvert.contains(currentAnnotatorID)) {
095                        ccpTA.setAnnotator(ccpAnnotator);
096                    }
097                } else {
098                    System.err.println("WARNING -- AnnotatorOverrider_AE: CCPTextAnnotation expected but instead got "
099                            + possibleAnnot.getClass().getName());
100                }
101            }
102    
103        }
104    
105    }
```
